# The Value of GeneXpert MTB/RIF for Detection in Tuberculosis: A Bibliometrics-Based Analysis and Review

**DOI:** 10.1155/2022/2915018

**Published:** 2022-10-15

**Authors:** Zhiyi Li

**Affiliations:** Laboratory Medicine, Nanan Hospital, Nanan, Quanzhou 362300, Fujian, China

## Abstract

With the continuous development of medical science and technology, especially with the advent of the era of precision diagnosis and treatment, molecular biology detection technology is widely valued and applied as an aid to early diagnosis of tuberculosis. The GeneXpert *Mycobacterium tuberculosis* Branching (MTB) technology is a suite of semi-nested real-time fluorescent quantitative PCR in vitro diagnostic technologies developed by Cepheid Inc. It targets the rifampicin resistance gene, rpoB, and can detect both MTB and resistance to rifampicin within 2 h. This review analyzed the papers related to GeneXpert using bibliometric software CiteSpace and Bibliometrix. A total of 151 articles were analyzed, spanning from 2011 to 2021. This bibliometrics-based review summarizes the history of the development of GeneXpert in tuberculosis diagnosis and its current status. Contributions of different countries to the topic, journal analysis, key paper analysis, and clustering of keywords were used to analyze this topic.

## 1. Introduction

Bacteriological and immunological detection techniques for tuberculosis require long detection times with poor sensitivity or specificity, which limit the early diagnosis and treatment of tuberculosis. In particular, smear-negative pulmonary tuberculosis, extrapulmonary tuberculosis, and *Mycobacterium tuberculosis* (MTB) latent infection lack typical clinical manifestations or imaging features. Fewer differential diagnostic techniques have led to a more complex situation in tuberculosis prevention and control. With the continuous development of medical science and technology, especially with the advent of the era of precision diagnosis and treatment, molecular biology detection technology has been widely used in the early diagnosis of tuberculosis. Molecular biology detection technology has the advantages of accuracy, high efficiency, and high throughput, bringing new light to the diagnosis and treatment of tuberculosis and the prevention and control of the epidemic.

MTB nucleic acid amplification techniques usually target IS6110, rpoB, gyrB, IS1081, culture filtrate protein 10 (CFP-10), etc. and are amplified and detected by PCR. In 2017, China published the standard WS288-2017 Tuberculosis Diagnosis, which includes a positive MTB nucleic acid test as one of the diagnostic criteria for tuberculosis. The advent of the GeneXpert MTB/RIF (GeneXpert) cassette test represents a breakthrough in molecular biology of tuberculosis. The GeneXpert assay kit was developed by Cepheid Inc. for use with the GeneXpert instrument. The technique is based on multiplexed semi-nested real-time fluorescence quantitative PCR to detect the MTB rifampicin resistance determining region rpoB gene. It detects the presence of MTB and its resistance to rifampicin directly from the patient's fresh or frozen sputum within 2 h. Studies have shown that the sensitivity of the GeneXpert test is 61.8% to 85.0% and the specificity is 98% to 99% for the examination of respiratory specimens from patients with pulmonary tuberculosis [1–3]. It has a sensitivity of 85% for the detection of sputum specimens from MTB/HIV co-infected patients [4]. It has a sensitivity of 98% for the detection of sputum specimens from patients with sputum smear-positive tuberculosis [4]. It has a sensitivity of 67% for sputum specimens from patients with sputum smear-negative tuberculosis [4]. The use of GeneXpert in the detection of smear-positive TB has been widely recognized. For suspected MTB/HIV dual infection, the World Health Organization recommends GeneXpert as the preferred test [5]. However, it has also been shown that GeneXpert is less sensitive in detecting specimens with low bacterial content, limiting its use in the detection of smear-negative tuberculosis and extrapulmonary tuberculosis patients [6].

To further improve the sensitivity of the GeneXpert assay, Cepheid has introduced the Xpert Ultra, a second-generation GeneXpert assay system. The assay system has two different multicopy amplification targets (IS6110 and IS1081) and a larger DNA reaction chamber (from a 25 *μ*L system in GeneXpert to a 50 *μ*L system in XpertUltra) [2]. XpertUltra also incorporates a full suite of nucleic acid amplification and a faster thermal cycle, which enables detection down to 16 CFU/mL compared to 114 CFU/mL for GeneXpert. In a recent prospective study [7], XpertUltra and GeneXpert tested sputum specimens from 137 smear-negative but culture-positive tuberculosis patients with sensitivities of 63% and 46%, respectively. It tested 115 culture-positive sputum specimens from patients with MTB/HIV co-infection with sensitivities of 90% and 77%, respectively. The overall specificity of the XpertUltra and GeneXpert assays was 96% and 98%, respectively. XpertUltra has a slightly lower specificity for relapsed patients compared to primary tuberculosis patients, but its sensitivity in detecting rifampicin resistance is consistent with that of GeneXpert. Currently, GeneXpert and XpertUltra are widely used in dozens of countries.

In recent years, many scholars have worked on the use of GeneXpert for the diagnosis of extrapulmonary tuberculosis. For example, bone tuberculosis lesions have a low number of MTBs in the lesions because they are at the end of the circulation. The traditional culture and smear method has faced the disadvantages of low positive rate and poor timeliness, and the early diagnosis of bone tuberculosis still faces challenges until now. In a study of 201 patients with suspected bone tuberculosis, GeneXpert detected eight more positive cases than the culture method, two of which were rifampicin-resistant patients [8]. Tuberculous pleurisy is the second most common form of extrapulmonary tuberculosis, and the sensitivity and specificity of biochemical, immunologic, and bacteriologic assays are low in ancillary laboratory tests. GeneXpert showed high sensitivity (90.0% and 72.0%) and specificity (100.0% and 100.0%) [9]. In addition, GeneXpert has been used in both urinary tract tuberculosis and tuberculous meningitis. To date, there have been a series of reviews and reviews on the value of the application of GeneXpert. For example, Brown et al. [10] summarized the use of GeneXpert in low and middle-income countries. Sagili et al. [11] used a review to analyze the relationship between cost and effectiveness of GeneXpert in the diagnosis of tuberculosis. However, no bibliometrics-based review is available to date. In this work, bibliometrics was used in a statistically based analysis of the use of GeneXpert in tuberculosis. Bibliometrics is a quantitative analysis of the literature that provides an understanding of a topic by analyzing the interrelatedness of information in different sections of a paper (e.g., title, keywords, authors, and reference). This analysis technique has been widely used in recent years for systematic reviews of different topics [12–18]. Bibliometric analyses on tuberculosis have focused on the development of the entire field [19–22]. However, the bibliometric analyses of GeneXpert have not been conducted. Therefore, we have paid special attention to the development of GeneXpert techniques in tuberculosis in this bibliometric work.

## 2. Material and Data Cleaning

Two kinds of bibliometrics software have been used in this systematic literature review. CiteSpace, developed by Dr. Chaomei Chen, a professor at the Drexel University School of Information Science and Technology [23–26], has been used in this work. CiteSpace 6.1R2 was used to calculate and analyze all documents. In addition, Bibliometrix package of R was used to perform some additional scientometric analysis [27].

**Figure 1:**
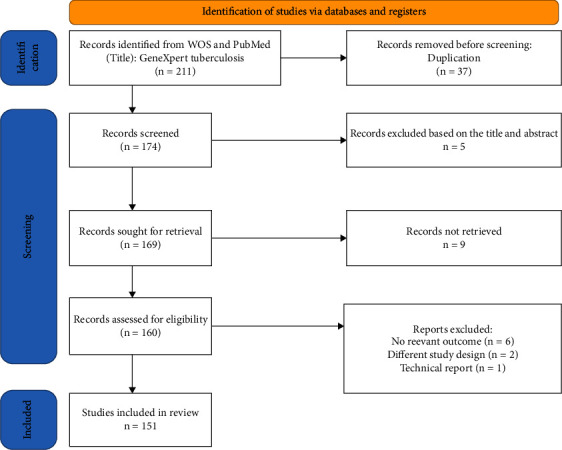
The Preferred Reporting Items for Systematic Reviews and Meta-Analyses (PRISMA) for this study.

Core collection on Web of Science and PubMed has been selected as a database to assure the integrity and academic quality of the studied material. “GeneXpert tuberculosis” has been used as a “Title.” The retrieval period was indefinite, and the date of retrieval was December, 2021. 151 articles were retrieved, spanning from 2011 to 2021 (Supporting Information). The detailed systematic literature search (Preferred Reporting Items for Systematic Reviews and Meta-Analyses, PRISMA) is shown in Figure 1.

## 3. Developments in the Research Field

### 3.1. Literature Development Trends

**Figure 2:**
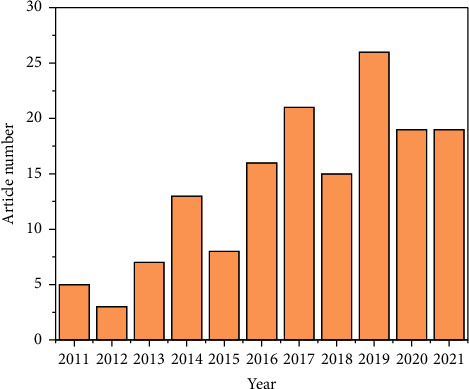
Annual publications from 2011 to 2021 searched in WOS and PubMed about the use of GeneXpert in tuberculosis.

The literature search for this work spanned from 2011 to 2021 and included a total of 151 papers. Among them, article, editorial material, letter, meeting abstract, and review are 109, 2, 7, 31, and 2, respectively.  Figure 2 shows the process of annual publication of papers on this topic. It can be seen that although the number of papers is not always increasing, the overall trend is increasing, with an annual growth rate calculated to be 14.44%. 151 papers include a total of 797 authors. Only 7 papers were published by a single author. The average number of authors per paper was 5.28. International collaborations were included in 23.18% of all papers. All papers contained 201 author keywords and 20732 citations.

### 3.2. Journals and Cited Journals

Table 1 shows the top 7 journals in terms of number of publications in this topic. The most published journal is the multidisciplinary journal PLOS One. The second most published journal is Journal of Clinical Microbiology. In addition, journals related to respiratory and infection also play important roles.

**Figure 3:**
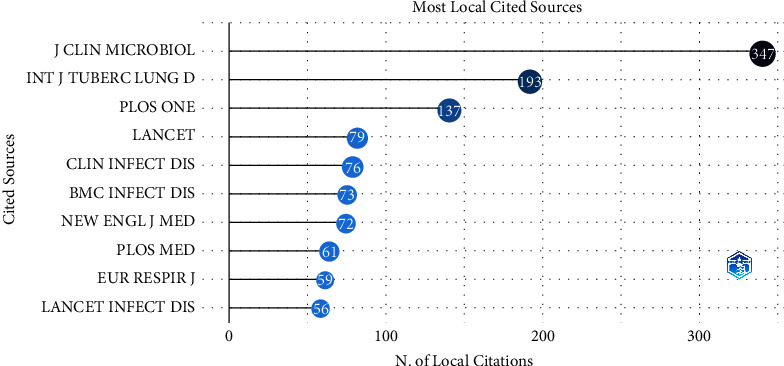
Top 10 cited journals on the GeneXpert in tuberculosis.

The contribution of different journals in a topic is measured not only by the number of papers it published but also by the frequency of citations of this journal in papers on this topic.  Figure 3 shows the top 10 cited journals on the GeneXpert in tuberculosis. The Journal of Clinical Microbiology, ranked second in Table 1, shows a definite dominance in Figure 2 with a total of 347 located citations. PLOS One, ranked first in Table 1, is ranked third in Figure 2, with a total of 137 located citations. In addition, the European Respiratory Journal and BMC Infectious Diseases, both of which are listed in Table 1, also appear in Figure 2, representing their publication of papers with significant impact in this topic. Some of the other important journals that appear in Figure 2 fall into two main categories. The first includes journals directly related to tuberculosis, such as the International Journal of Tuberculosis and Lung Disease. The other category includes the most prestigious journals in the medical field, such as the Lancet and the New England Journal of Medicine.

**Figure 4:**
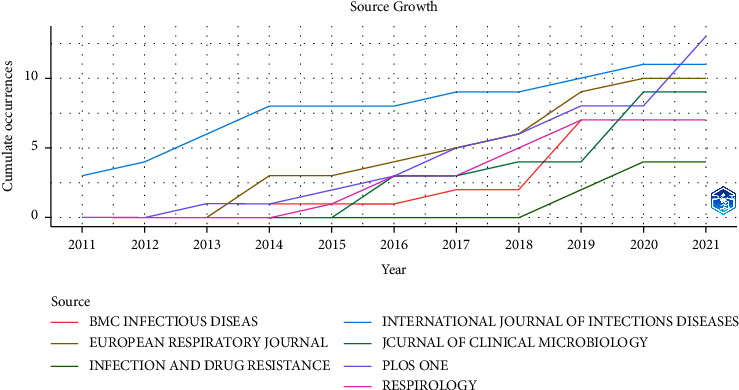
Accumulated journal dynamics of top 7 journals with highest publication number.

Figure 4 shows the chronology of papers published in this topic by the seven journals in Table 1. It can be seen that International Journal of Infectious Diseases was the first journal to publish a paper on this topic. In contrast, Infection and Drug Resistance did not publish a paper on this topic until 2018. Other journals started to engage in this topic between 2013 and 2016.

### 3.3. Geographic and Author Distribution

**Figure 5:**
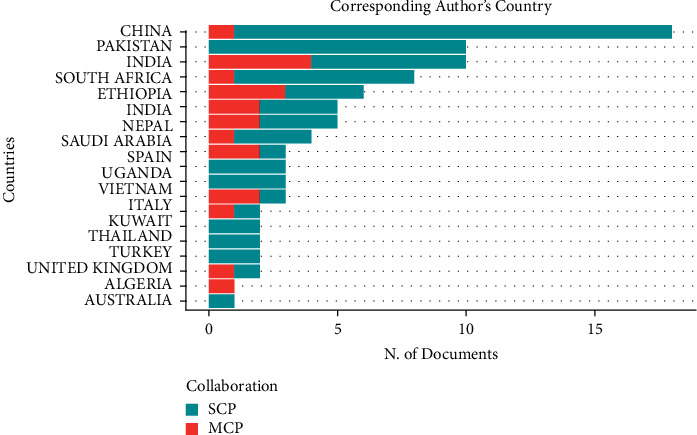
Top 20 corresponding author countries.

Table 2 shows the level of participation of different countries in this topic. It can be seen that Pakistan is the most engaged country in this topic. In addition, China, USA, and South Africa all have a frequency of more than 40 times. From this result, it can be guessed that the level of participation in this topic depends on two main factors. The first group of countries includes traditionally large scientific countries, such as USA and China. Another reason is that the diagnosis of TB has variability in countries with different levels of health and climate. Therefore, some countries will present an active stance in this topic. Table 2 shows the level of participation of different countries and does not represent that these countries present a leadership position in this topic. Therefore, we further analyzed the countries of the corresponding authors.  Figure 5 shows the distribution of corresponding authors by country in the papers on this topic. It can be seen that the largest number of corresponding authors is from China. The second highest is Pakistan. Although there are some differences between the results in Figure 4 and the order in Table 2, they basically match. This represents that these countries in Table 2 do play an important role in the development of this topic.

**Figure 6:**
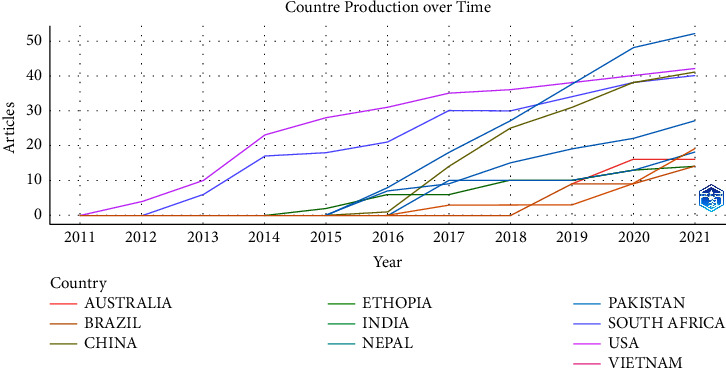
Countries' production over time.

Figure 6 shows the publication chronology of different countries for this topic. It can be seen that USA is the first country to participate in this topic, probably because GeneXpert is developed by a US company. South Africa started to participate in this topic in 2012 and has been active since then. In contrast, both Pakistan and China did not publish papers on this topic until the beginning of 2016. Among the countries in Table 2, Vietnam was the latest to participate in this topic. However, it is worth noting that this bibliometric analysis only retrieved core collections in WOS. Therefore, this does not necessarily represent the exact time of participation of these countries in this topic.

**Figure 7:**
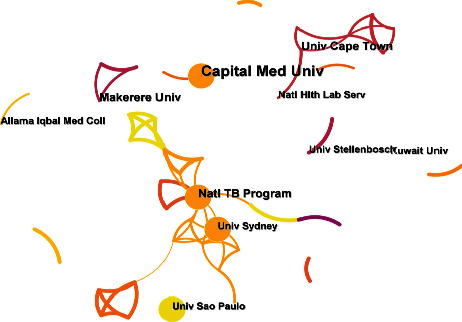
Affiliation cooperation network of published papers for GeneXpert in tuberculosis.

Table 3 shows the top 10 affiliations involved in this topic. South African institutions play an important role, such as the University of Cape Town, Wits University, and Stellenbosch University.  Figure 7 shows the network of collaboration between different institutions. As can be seen from the figure, there are 2 main collaborative networks formed by this topic. The first collaborative network is relatively small and is led by the University of Cape Town and the National Institute of Respiratory Diseases. The second collaborative network consists of a range of institutions, led by the National Tuberculosis Programme and the University of Sydney.

## 4. Keywords and Key Publication Analysis

**Figure 8:**
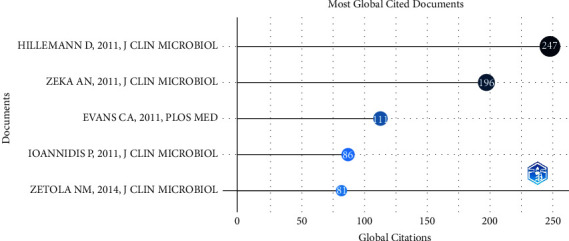
Top 5 most global cited documents on GeneXpert in tuberculosis.

Figure 8 shows the top 5 most globally cited documents. These papers received a lot of attention and citations after publication. The paper published by Hillemann et al. [28] in 2011 has received 247 citations to date. They tested 521 non-respiratory specimens from the National Reference Laboratory for *Mycobacterium avium* in Germany using GeneXpert. The test results were compared with solid culture methods. Of these, GeneXpert yielded no results for 20 samples. The ensemble sensitivity and specificity of GeneXpert were 77.3% and 98.2%, respectively. Notably, GeneXpert can reach 100% sensitivity for urine and feces. Zeka et al. [29] investigated the performance of GeneXpert against rifampicin resistance. This study included 253 lung specimens and 176 extrapulmonary specimens. The results indicated a sensitivity of 100% and 68.6% for smear-positive and smear-negative specimens, respectively, in pulmonary specimens. Sensitivity was lower for extrapulmonary specimens with 47.7% for smear-negative specimens. Based on the results, they concluded that GeneXpert is a simple method that operators can make with only simple training. Evans' 2011 article [30] on GeneXpert is a review of this technology. This perspective argues that this technology has great potential but must be seen in the shameful context of the nearly 2 million people who die from TB each year. The purchase of GeneXpert should not become a part of the current financial crisis coupled with a huge unmet need in other health areas. Ioannidis et al. [31] systematically measured pulmonary specimens, extrapulmonary specimens, and microscopically negative specimens. They concluded based on their results that GeneXpert is very effective for the diagnosis of tuberculosis and identification of rifampicin-resistant strains in sputum smear-negative specimens. Zetola et al. [32] specifically focused on the feasibility of GeneXpert in the detection of mixed MTBC infections. The results of the study showed an increased false-negative rate of GeneXpert for detecting rifampicin resistance in mixed MTBC infections. Therefore, GeneXpert results may require further confirmation in areas where mixed infections are common.

Among the most locally cited documents, four of the top five papers matched those in Figure 7, except for one review published by Bunsow et al. [33]. They used data from 595 clinical samples from a hospital in Madrid, Spain, containing 305 non-respiratory samples and 290 respiratory samples. The techniques compared included gold amine smears, routine cultures, and the MGIT SIRE (BD) drug phenotypic susceptibility test, yielding a total of 81 cases of *Mycobacterium tuberculosis*. 71 of these cases were detected positively by GeneXpert. They concluded that GeneXpert is an accurate, easy-to-apply, and rapid detection technique, especially for sputum-coated positive respiratory samples.

Table4 shows the top 5 of the most local cited references on GeneXpert in tuberculosis. These papers largely set the stage for the development of this topic. Boehme et al. [34] evaluated the performance of GeneXpert. A total of 1730 samples from patients with suspected drug-sensitive or multidrug-resistant tuberculosis were included in this study. The population included Peru, Azerbaijan, South Africa, and India. A single direct GeneXpert test confirmed 551 of 561 smear-positive tuberculosis cases and 124 of 171 smear-negative tuberculosis cases. Thus, GeneXpert can detect tuberculosis and rifampicin resistance in less than 2 h. Zifodya et al. [1] compared the diagnostic accuracy of Xpert Ultra and Xpert MTB/RIF in the detection of pulmonary tuberculosis and rifampicin resistance in presumptive adult tuberculosis patients. Helb et al. [35] published the first report on MTB/RIF of the Xpert system in 2010. Since our search was performed with GeneXpert as a keyword and only the title was searched, this paper did not appear in the results. Boehme et al. [36] also conducted large-scale tests to validate the feasibility, diagnostic accuracy, and effectiveness of GeneXpert. The effect of GeneXpert was also validated by Marlowe et al. [37]. They had a smaller sample size.

**Figure 9:**
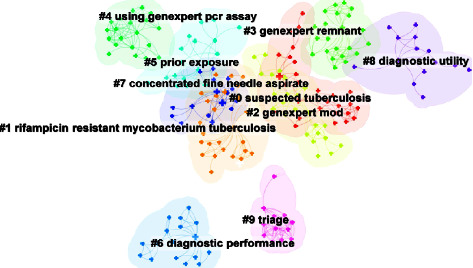
Grouping of keywords for GeneXpert in tuberculosis.

Table 5 and Figure 9 show the clustering analysis of keywords. Tuberculosis is a specific type of inflammatory disease. It is the largest global threat of death caused by a single infectious agent. Tuberculosis can be divided into pulmonary and extrapulmonary tuberculosis, of which about 1/5 are extrapulmonary tuberculosis, which can cause infection and even disease in close contacts by expelling microdroplets containing *Mycobacterium tuberculosis* through coughing and sneezing. The exact pathogenesis of tuberculosis is still unknown and consists mainly of infection by *Mycobacterium tuberculosis*, a series of immune responses triggered by the infection in the host, and host disease. In most developing countries, the diagnosis of tuberculosis is still made using relatively outdated tools and methods, including chest radiographs and sputum smear microscopy. The disadvantages of these diagnostic tools are more obvious, such as longer time consumption, higher false-negative rate, lower positive rate, and inability to promote their application in grassroots organizations on a large scale. These clinical primary health organizations, in turn, have the functional task of making the vast majority of initial diagnoses. Some patients with extrapulmonary tuberculosis do not have a range of clinical symptoms due to *Mycobacterium tuberculosis*, but rather a range of clinical symptoms due to respiratory infections caused by other pathogenic bacteria. There are also some general practitioners and non-tuberculosis specialists who are able to make routine diagnoses but lack a certain amount of expertise in tuberculosis, resulting in longer diagnosis times, more frequent referrals, and other delays in the diagnosis and treatment of tuberculosis. In 2011, the World Health Organization strongly recommended the GeneXpert MTB/RIF automated molecular identification of *Mycobacterium tuberculosis* and rapid diagnosis of rifampicin resistance as the initial diagnostic method involving multidrug-resistant tuberculosis or AIDS-associated tuberculosis. The GeneXpert assay has gained widespread interest because of its high sensitivity and specificity and its ease and speed of use. The GeneXpert assay is a landmark in the study of tuberculosis.

## 5. Conclusion

The GeneXpert assay has been widely reported in recent years as a type of nucleic acid assay. The GeneXpert test is more sensitive and accurate than traditional smear and culture methods and can detect *Mycobacterium tuberculosis* even in the presence of negative sputum smears. It therefore enables early intervention in tuberculosis patients to prevent serious consequences of tuberculosis disease without delaying the optimal treatment of first diagnosed patients. In areas with a high prevalence of TB, the GeneXpert test is important for rapid diagnosis of drug-resistant tuberculosis and for guiding rational clinical use of drugs.

GeneXpert has shown higher sensitivity and specificity than traditional smear and culture in the diagnosis of extrapulmonary tuberculosis such as bone TB, tuberculosis pleurisy, and urological tuberculosis. It can also detect rifampicin resistance and make a preliminary determination of MTB or non-MTB in smear-positive patients. In addition, studies on the specific advantages of GeneXpert in terms of timeliness and its impact on the ultimate prognosis and survival of patients are expected. With the increasing number of cases of tuberculosis resistant to other first-line and second-line drugs, such as multidrug-resistant tuberculosis, GeneXpert is not yet able to detect them, so the existing system needs to be improved to cover more drug-resistant loci.

On the other hand, the high cost of testing makes it difficult to scale up these technologies in low and middle-income countries, where a large proportion of tuberculosis patients are located. Therefore, in contrast to the pursuit of technologies with high sensitivity, high specificity, and full automation, it is particularly important to develop molecular biology assays that are suitable for medically disadvantaged areas based on a comprehensive assessment of technical performance and affordability. In addition to economic constraints, these areas often have limited access to timely medical services, especially for molecular biology testing, which requires high laboratory conditions. Therefore, GeneXpert not only needs to improve efficiency and reduce costs, but also needs to develop a rapid detection system to meet the immediate detection needs in remote areas.

## Figures and Tables

**Table 1:** Top 7 journals that published articles on the GeneXpert in tuberculosis.

Journal	Articles
PLOS One	14
Journal of Clinical Microbiology	11
European Respiratory Journal	10
International Journal of Infectious Diseases	9
BMC Infectious Diseases	8
Respirology	7
Infection and Drug Resistance	4

**Table 2:** Top 10 countries' scientific production on the GeneXpert in tuberculosis.

Country	Frequency
Pakistan	52
USA	42
China	41
South Africa	40
India	28
Brazil	20
Nepal	18
Australia	16
Ethiopia	15
Vietnam	14

**Table 3:** Top 10 affiliations that participated in the GeneXpert in tuberculosis.

Affiliation	Articles
University of Cape Town	13
Capital Medical University	12
University California San Francisco	8
Makerere University	7
National Health Laboratory Service	6
Universidade de São Paulo	6
National Institute of Respiratory Diseases	6
Stellenbosch University	5
The University of Sydney	5
Wits University	5

**Table 4:** Top 5 of the most local cited references on GeneXpert in tuberculosis.

Cited reference	Citations
Boehme CC, 2010, new engl J med, V363, P1005, DOI 10.1056/NEJMOA0907847	[47]
Steingart KR, 2014, cochrane Db syst rev, DOI 10.1002/14651858.CD009593.PUB3, 10.1002/14651858.CD009593.PUB2	[34]
Helb D, 2010, J clin microbiol, V48, P229, DOI 10.1128/jcm.01463–09	[32]
Boehme CC, 2011, lancet, V377, P1495, DOI 10.1016/s0140-6736(11)60438–8	[27]
Marlowe EM, 2011, J clin microbiol, V49, P1621, DOI 10.1128/jcm.02214–10	[22]

**Table 5:** Knowledge clusters in the field of GeneXpert in tuberculosis on keyword co-occurrences for each cluster.

Cluster ID	Size	Silhouette	Keywords	References
0	28	0.928	Assay; resistance; complex; polymerase chain reaction	[28, 38–46]
1	27	0.906	Diagnosis; accuracy; GeneXpert MTB/RIF; bactec mgit 960; feasibility	[39, 47–54]
2	25	0.931	Performance; xpert MTB/RIF assay; disease; rifampinresistance	[55–61]
3	22	0.969	Drug resistance; mutation; epidemiology	[30, 32, 62–64]
4	20	DNA	Identification; GeneXpert assay; DNA	[65, 66]
5	18	0.890	Microscopy; children; HIV	[67–70]
6	17	0.988	Pulmonary tuberculosis; prevalence; impact	[71–78]
7	16	0.890	Xpert MTB/RIF; rifampin resistance; rapid diagnosis; specimen	[47, 58, 59, 79–86]
8	14	0.850	Extrapulmonary tuberculosis; culture; GeneXpert MTB	[74, 79, 87, 88]
9	12	0.957	Cost effectiveness; molecular diagnostic technique; acidamplification test	[89]

## Data Availability

The data supporting this review are from previously reported studies and datasets, which have been cited. The processed data are available in the supplementary files.

## Supplementary Materials

Supplementary Materials The supporting information includes the original data used in the bibliometric analysis.
